# Hepatitis E genotype 3 genome: A comprehensive analysis of entropy, motif conservation, relevant mutations, and clade-associated polymorphisms

**DOI:** 10.3389/fmicb.2022.1011662

**Published:** 2022-10-06

**Authors:** Milagros Muñoz-Chimeno, Vanessa Rodriguez-Paredes, Maira Alejandra García-Lugo, Ana Avellon

**Affiliations:** ^1^Hepatitis Unit, National Center of Microbiology, Carlos III Institute of Health, Madrid, Spain; ^2^Alcalá de Henares University, Madrid, Spain; ^3^CIBERESP Epidemiology and Public Health, Madrid, Spain

**Keywords:** hepatitis E virus, HEV, domains, polymorphisms, entropy, genome, genotypes, motif

## Abstract

Hepatitis E virus genotype 3 (HEV-3) is an EU/EEA emergent zoonosis. HEV-3 clades/subtypes have been described. Its genome contains ORF1, which encodes nonstructural proteins for virus replication, ORF2, the capsid protein, and ORF3, a multifunctional protein involved in virion pathogenesis. The study aims with respect to HEV-3 are to: (1) calculate genome entropy (excluding hypervariable region); (2) analyze the described motifs/mutations; (3) characterize clade/subtype genome polymorphisms. Seven hundred and five sequences from the GenBank database were used. The highest entropies were identified in zoonotic genotypes (HEV-3 and HEV-4) with respect to HEV-1 in X domain, RdRp, ORF2, and ORF3. There were statistically significant differences in the entropy between proteins, protease and ORF3 being the most variable and Y domain being the most conserved. Methyltransferase and Y domain motifs were completely conserved. By contrast, essential protease H581 residue and catalytic dyad exhibited amino acid changes in 1.8% and 0.4% of sequences, respectively. Several X domain amino acids were associated with clades. We found sequences with mutations in all helicase motifs except number IV. Helicase mutations related to increased virulence and/or fulminant hepatitis were frequent, the 1,110 residue being a typical HEV-3e and HEV-3f-A2 polymorphism. RdRp motifs III, V, VII also had high mutation rates. Motif III included residues that are polymorphisms of HEV-3e (F1449) and HEV-3 m (D1451). RdRp ribavirin resistance mutations were frequent, mainly 1479I (67.4, 100% in HEV-3efglmk) and 1634R/K (10.0%, almost 100% in HEV-3e). With respect to ORF2, 19/27 neutralization epitopes had mutations. The S80 residue in ORF3 presented mutations in 3.5% of cases. Amino acids in the ORF3-PSAP motif had high substitution rates, being more frequent in the first PSAP (44.8%) than in the second (1.5%). This is the first comprehensive analysis of the HEV-3 genome, aimed at improving our knowledge of the genome, and establishing the basis for future genotype-to-phenotype analysis, given that viral features associated with severity have not been explored in depth. Our results demonstrate there are important genetic differences in the studied genomes that sometimes affect significant viral structures, and constitute clade/subtype polymorphisms that may affect the clinical course or treatment efficacy.

## Introduction

Hepatitis E virus (HEV) is the most common cause of acute viral hepatitis worldwide (Aslan and Balaban, 2020), and is also responsible for severe chronic infections in immunocompromised patients. The number of EU/EEA-acquired infections has been increasing over the last decade (Adlhoch et al., 2016). HEV is a member of the *Hepeviridae* family and the *Orthohepevirus* genus, whose group A includes zoonotic viruses that infect humans, pigs, rabbits, wild boars and camels, among others (Purdy et al., 2017). Eight genotypes have been described so far, five of which can infect humans (HEV 1, 2, 3, 4, and 7; Smith et al., 2016). HEV genotype 3 (HEV-3) is a zoonosis. Pigs are its most important reservoir and it is transmitted through the consumption of raw or undercooked meat (Garcia et al., 2019). According to Smith et al. (2020), HEV-3 is further divided into subtypes 3a, 3b, 3c, 3e, 3f, 3g, 3h, 3i, 3j, 3k, 3l, 3m and some unassigned sequences. Subtypes are grouped in two major clades: HEV-3efg and HEV-3abjkchilm (Smith et al., 2016). The latter clade is further divided into HEV-3abjk and HEV-3chilm. A recent study divided HEV-3f into three clusters (HEV-3f-A1, HEV-3f-A2 and HEV-3f-B), and HEV-3m into HEV-3m-A and HEV-3m-B, based on p-distance and phylogenetic analysis (Munoz-Chimeno et al., 2022).

The HEV genome is a 7.2-kb single-stranded positive-sense RNA molecule containing three partially overlapping open reading frames (ORF1, ORF2, and ORF3; Tam et al., 1991). HEV-1 was recently shown to have an additional reading frame (ORF4) that overlaps with ORF1 and is required to ensure correct HEV RNA polymerase function (Nair et al., 2016). In 1992, computational analysis of the non-structural polyprotein identified seven putative domains [methyltransferase, Y domain, putative papain-like cysteine protease, proline-rich hinge domain, X domain, putative RNA helicase and putative RNA polymerase (Koonin et al., 1992)].

HEV methyltransferase (**MTase**) is characterized by 5′-mRNA capping activity through guanyltransferase and guanine-7-methyltransferase activities (Kabrane-Lazizi et al., 1999; Magden et al., 2001). It also presents the four highly conserved sequence motifs described in the capping proteins of positive-strand RNA viruses (Rozanov et al., 1992). The A105H mutation in Mtase was associated with viremia decrease (Borkakoti et al., 2017) and F179S was associated with fulminant hepatitis (Mishra et al., 2013). The non-structural **Y domain** was initially found approximately 200 amino acid residues downstream of the MTase domain (Koonin et al., 1992). It contains critical conserved residues that have been shown to play a crucial role in virus replication (Parvez, 2017; Cao et al., 2018) and adaptation (Shafat et al., 2021). HEV protease is a putative papain-like cysteine protease (**PCP**) and although its functions are still under debate, molecular analysis of mutant replicons identified highly conserved cysteines and histidines that are essential for replication (Parvez, 2013), catalytic dyad (Parvez and Khan, 2014) and a Zn^2+^ binding site that is crucial for viral activity (Saraswat et al., 2019). The **HEV X domain** was classified in 2003 as the ADP-ribose-1″-monophosphatase of macrodomain protein family (Allen et al., 2003) and includes highly conserved residues that corroborate this classification (Parvez, 2015). In addition, C-terminal region presents residues whose interaction suggests a relevant role in the viral cycle (Anang et al., 2016), as well as Mg^2+^ and Zn^2+^ binding sites (Vikram and Kumar, 2018). The HEV X domain may play a role in viral replication and/or translation (Neuvonen and Ahola, 2009) and has been identified as a putative IFN antagonist *in vitro* (Nan et al., 2014). The putative RNA helicase (**Hel**) contains seven conserved motifs (I, Ia, II, III, IV, V, and VI) of the helicase’s superfamily 1 (Gorbalenya et al., 1989; Hall and Matson, 1999). Hel mutations have been associated with virus virulence and disease severity (Inoue et al., 2009; Takahashi et al., 2009; Devhare et al., 2014; Cao et al., 2018). **RdRp**, which is phylogenetically classified in supergroup III, catalyzes the RNA viral synthesis at several levels (Oechslin et al., 2022) and contains a highly conserved GDD motif, which constitutes a catalytic triad associated with the replicative activity (Koonin, 1991; Rehman et al., 2008). Several mutations in the RdRp region have been associated with adverse clinical outcomes (Debing et al., 2014, 2016; Borkakoti et al., 2016, 2017; Todt et al., 2016). **ORF2**, which contains three domains, designated S, M, and P, was initially considered to encode only the capsid protein (Robinson et al., 1998; Graff et al., 2008), but in recent years has been shown to present itself in different forms with multiple functions rather than just acting as a viral capsid (Ankavay et al., 2019). Several mutations are associated with reduced replication and infectivity (Cordoba et al., 2011). Two initiation codons have recently been described (Yin et al., 2018). ORF2-encoded protein is also responsible for the humoral immune response and therefore harbors antigenic properties (Khudyakov et al., 1994; Zhou et al., 2005) and neutralization epitopes (Gu et al., 2015; Ikram et al., 2018). **ORF3**, the smallest ORF, partially overlaps with the N-terminus ORF2 and is translated from a different reading frame. ORF3 is essential for HEV infection and is required for viral particle release (Graff et al., 2005; Yamada et al., 2009). Moreover, a reported motif within ORF3 protein has been shown to be required for membrane-associated HEV particle formation (Nagashima et al., 2011). Currently, HEV-ORF3 is thought to form an ion channel that is required for virion particle release from cells during infection (Ding et al., 2017). Similarly to ORF2, ORF3 has three distinct initiation codons (Graff et al., 2006).

This study aims to: (1) measure HEV-3, HEV-1 and HEV-4 genome entropy that has not yet been analyzed; (2) analyze conservation of HEV-3 genome functional motifs and mutations; and (3) identify and characterize HEV-3 subtype polymorphisms throughout the coding genome. The hypervariable region (HVR) was not analyzed in this study as this work has already been reported (Munoz-Chimeno et al., 2020).

## Materials and methods

### Sequence collection for genome analysis and sub-genotype assignment

HEV-3 genomes included in the analysis were retrieved from GenBank, 74 of which had previously been obtained in our laboratory (MZ289076-MZ289149; Munoz-Chimeno et al., 2022) and 439 were additional complete genomes retrieved from GenBank database in October 2021 (detailed in Supplementary material). In addition, 60 HEV-1 and 92 HEV-4 sequences from GenBank were used for entropy analysis (see Supplementary material). Reference sequences proposed by Smith et al. (2020) and Munoz-Chimeno et al. (2022) were used.

Sub-genotypes were checked by phylogenetic analysis as described elsewhere (Munoz-Chimeno et al., 2022).

### Alignment, translation and motif/position identification

Sequences were aligned using MAFFT-based alignment v.7 [MAFFT alignment and NJ/UPGMA phylogeny (cbrc.jp)]. Protein translation was carried out with the MEGA 7.0 software package.^1^ The hypervariable region was then removed from the alignment. The beginning and end of each ORF and protein were identified according to Koonin et al. and individual protein alignments were generated. Amino acids were numbered according to the position of each residue in each ORF (ORF numbering) according to the sequence with GenBank accession number KU513561. Additionally, in the case of ORF1, protein numbering (from 1 to the end of each protein) is set out in the text and Supplementary material. Previously reported relevant motifs or positions were localized by searching for their sequences in the alignments.

### Entropy analysis of the genome of HEV proteins

Shannon entropy, calculated with the Antigenic Variability ANAlyzer (AVANA) tool, was considered to be a measure of the variability of HEV protein sequences. Only sequences containing a valid amino acid at each position were used for the entropy analysis. Entropy was calculated by position first in the HEV-1, HEV-3 and HEV-4 genotype alignment, and second in the HEV-3 main clades (HEV-3abjk, HEV-3chilm and HEV-3efg). Levels of entropy were categorized as low (<0.03), intermediate (0.03–0.06) or high (>0.06).

### Statistics

Mean entropy in each protein and in each clade and protein was compared by ANOVA and *post hoc* Scheffe tests in IBM SPSS Statistics v.25.0. The threshold of significance was set at *p* < 0.05.

### Identification of polymorphisms associated with clades and sub-genotypes

To identify the polymorphisms associated with clades and subtypes, the percentages of each amino acid in each position by clade and subtype were calculated. To be considered polymorphisms and subtype/clade characteristic residues, the percentage of the specific amino acid should be at least 90% that in the subtype/clade.

## Results

### HEV genome entropy

**Figure 1:**
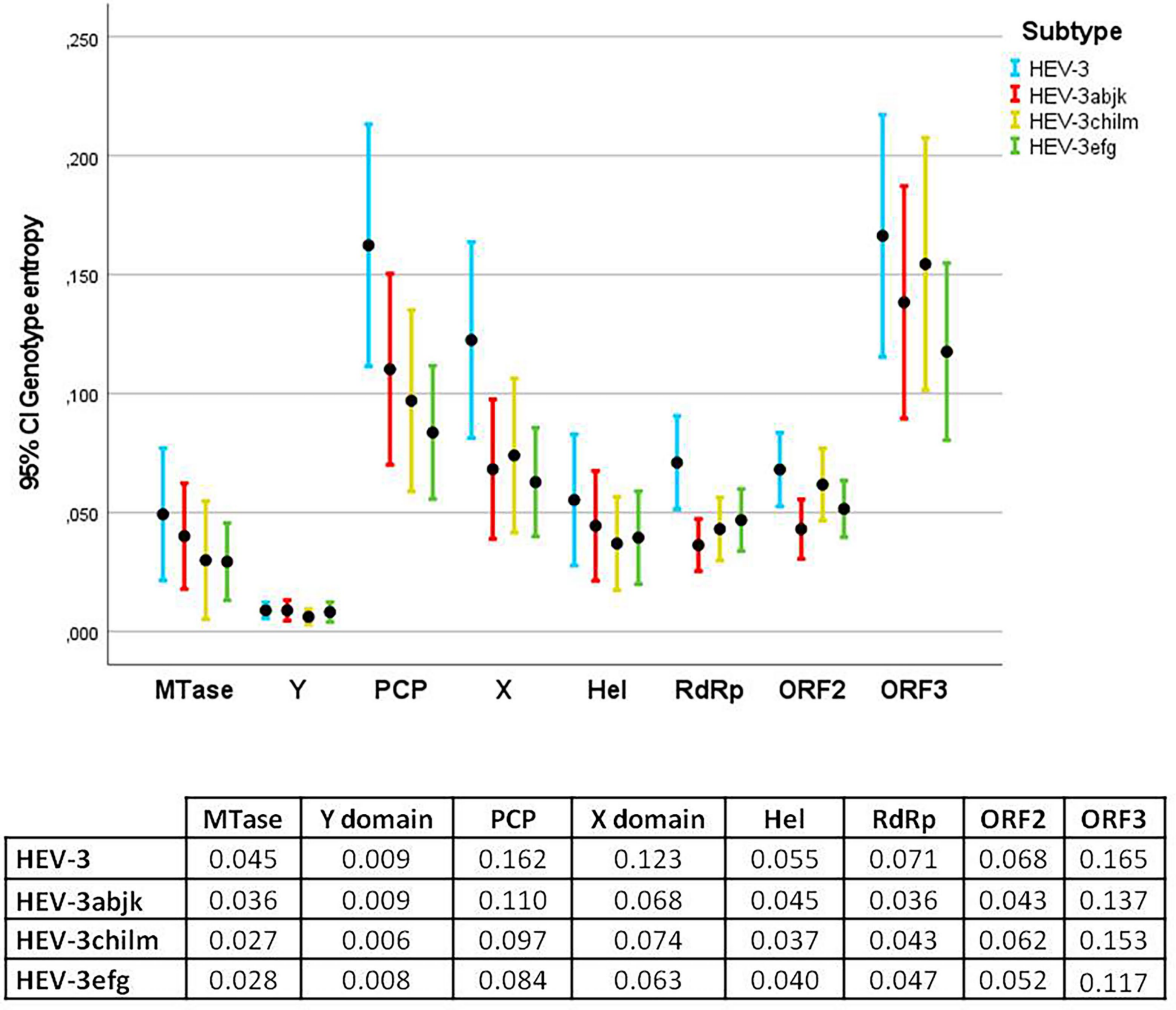
Error bar graph showing the mean entropy and 95% confidence interval (CI) for each protein in HEV-3, HEV-3abjk, HEV-3chilm, and HEV-3efg. The table below shows the average entropies of each protein. PCP and ORF3 exhibited statistically significant differences compared with MTase, Y domain, Hel, RdRp, and ORF2.

**Figure 2:**
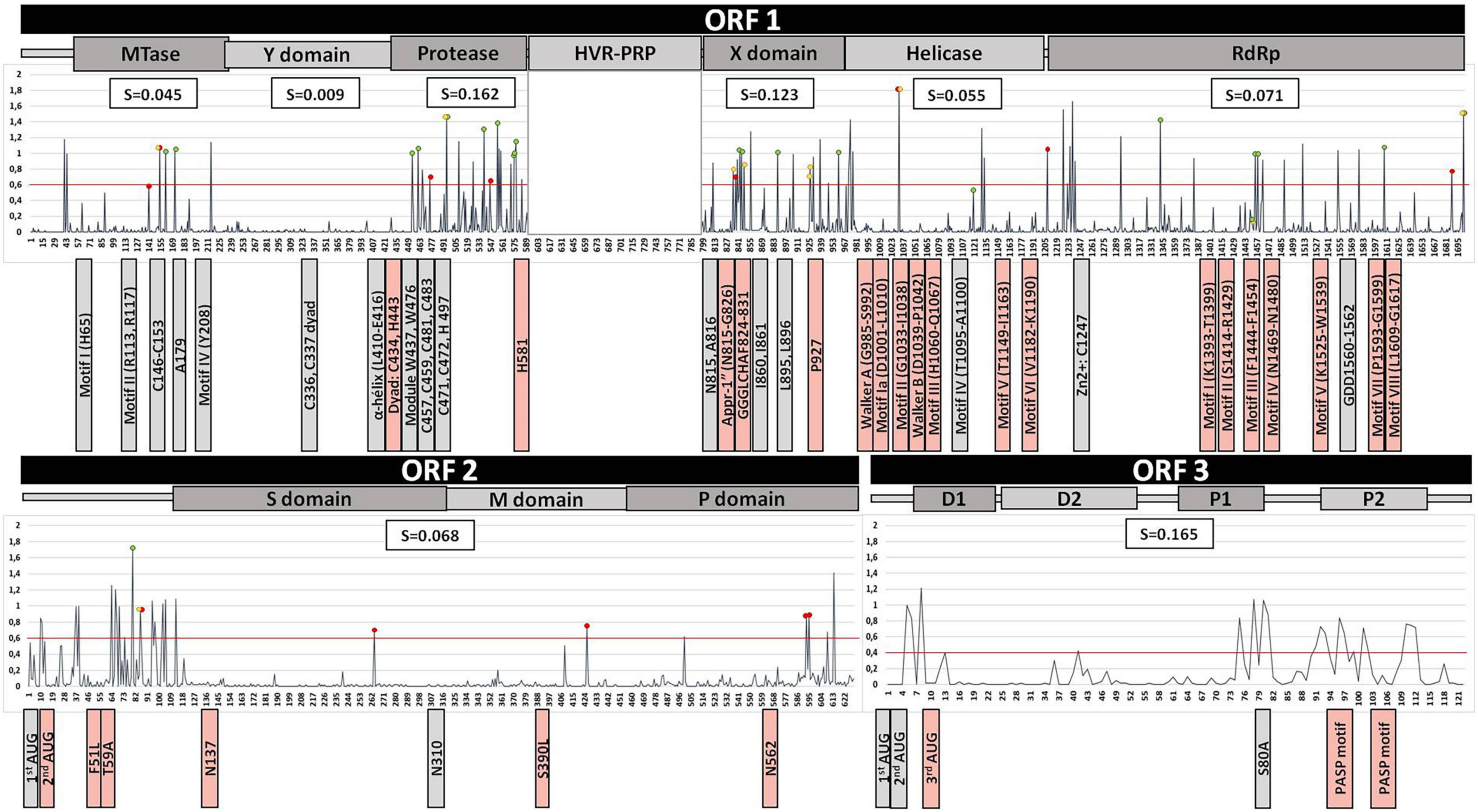
Summary of the HEV-3 genome analysis, excluding the HVR region, based on the alignment of 533 sequences. Line chart shows the entropy (S) of each position throughout the HEV genome. Red, yellow, and green dots indicate the positions of polymorphisms associated with the HEV-3abjk, HEV-3chilm, and HEV-3efg clades, respectively. The red line shows an entropy level of 0.6. The average entropy of each protein is shown in the white boxes above the chart. Previously reported motifs are indicated below the chart as boxes next to their position (grey, fully conserved motifs; light-red, mutated motifs).

Average entropies by protein/ORFs (excluding HVR) in the HEV-3 genome and by clade are shown in Figures 1, 2. Several statistically significant differences were observed. The PCP and ORF3 showed higher average entropy (0.162 and 0.165, respectively) than MTase, the Y domain, Hel, RdRp and ORF2 (*p* < 0.05). The Y domain had the lowest entropy in all clades (0.009) with respect to the PCP, X domain and ORF3 (0.162, 0.123, and 0.165, respectively). There were no statistically significant differences among the HEV-3 clades. The entropy at several positions along the HEV-3 genome was ≥0.6 (Table 1). PCP and ORF3 were proteins with a higher percentage of high-entropy positions (10.7% and 12.3% of total protein, respectively). ORF2 was the protein with the highest number of high-entropy positions (*n* = 24). By contrast, the Y domain did not have any high-entropy positions. The most variable positions in the X domain were 906 and 938. High-entropy positions were noted in all HEV-3 clades, the HEV-3chilm clade containing more of these than the other clades.

**Table 1:** Amino acid positions with entropy ≥0.6 throughout the genome.

Protein/ORF	HEV-3 amino acid positions with entropy ≥0.6	*N* entropy ≥0.6	Total AA in the protein	% entropy ≥0.6
MTase	141, 154, 161, 172, 215	5	185	2.7
Y domain	–	0	218	0.0
PCP	454, 461, 466, 475, 495, 509, 526, 539, 546, 555, 557, 559, 571, 575, 576, 577, 584	17	159	10.7
X domain	811, 835, 838, 840, 843, 845, 848, 856, 888, 906, 926, 930, 938, 948, 960	15	172	8.7
Helicase	972, 973, 974, 977, 1,032, 1,130, 1,133	7	233	3.0
RdRp	1,227, 1,232, 1,235, 1,238, 1,241, 1,295, 1,342, 1,382, 1,455, 1,458, 1,464, 1,489, 1,511, 1,553, 1,578, 1,608, 1,688, 1702	18	487	3.7
ORF2	10, 11, 36, 37, 39, 64, 67, 68, 70, 74, 80, 86, 95, 97, 103, 105, 113, 264, 426, 500, 593, 595, 609, 614	24	629	3.8
ORF3	5, 6, 8, 75, 78, 80, 81, 92, 93, 96, 97, 101, 110, 111, 112	15	122	12.3

Numbering is set according to ORF.

**Figure 3:**
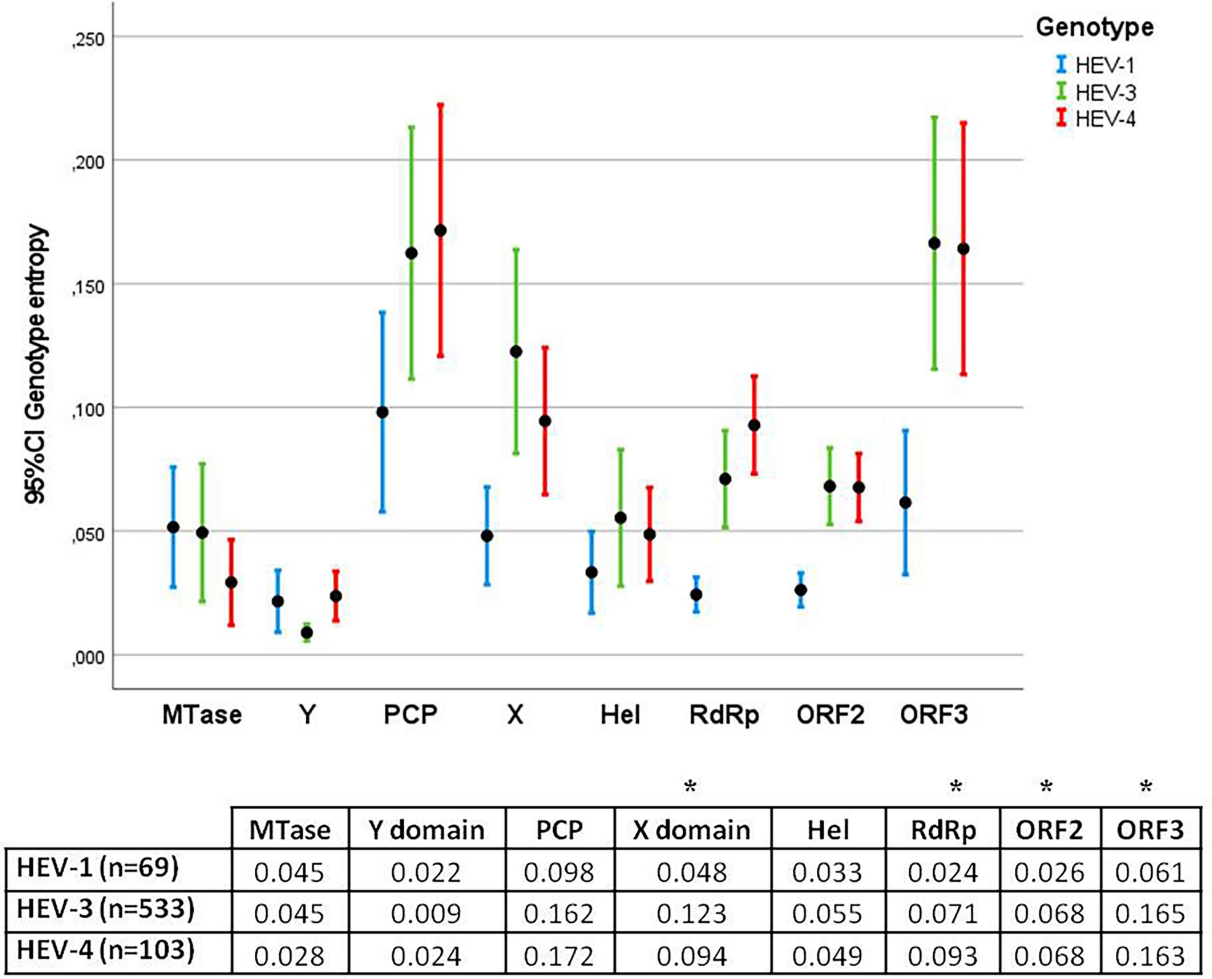
Error bar graph showing the mean entropy and 95% confidence interval (CI) for each protein in HEV-1, HEV-3, and HEV-4. The table below shows the average entropies of each protein. Statistically significant differences between genotypes were observed in the X domain, RdRp, ORF2 and ORF3 (*).

Entropy was also assessed by genotype (HEV-1, HEV-3, and HEV-4) and protein (Figure 3). The highest entropies were identified in genotypes HEV-3 and HEV-4 (zoonotic genotypes) relative to HEV-1 (main non-zoonotic genotype) in the X domain, RdRp, ORF2 and ORF3 (*p* < 0.05).

### Analysis of previously reported HEV genome motifs and mutations

Results of the ORF1 analysis of the HEV-3 genome are summarized in Table 2 and Figure 2. Previously reported motifs in the **Mtase** and **Y domain** are fully conserved, while an Mtase position known to be associated with fulminant hepatitis in HEV-1 (F179) was found to differ in the HEV-3 sequences analyzed, the wild type being 179A. Neither the palmitoylation site nor the α-helix of the **Y domain** had substitutions. **PCP** motifs associated with protease activity and structural integrity (C and W residues, respectively) were all conserved, with the exception of C434 (0.2% C434S). Notably, mutations of Y, Q and R were observed in H581 (1.9%) and of H in Y443 (0.2%). Reviewing the motifs and known mutations of the **X domain** revealed a change in G826D in 0.2% of sequences, and mutations in the metal binding sites G826D, L827I, and H829LR (0.9%) and P927L (14.1%), which is characteristic of the HEV-3c subtype. Analysis of **Hel** revealed mutations in every described motif except motif IV. The others exhibited changes ranging from 0.2% to 6.4%. Two sequences had at least two mutations in the Walker A motif. The two mutations reported to be associated with fulminant hepatitis in HEV-1 (L1110F and V1120I) were found in 0% and 51.9% of cases, respectively. Nevertheless, L1110MQRSV was frequently found (9.9%), 1110M being characteristic of the HEV-3e and HEV-3f-A2 clades. The mutation related to increased virulence in HEV-3 (V1208A) was present in 2.4% of the sequences studied and found to be characteristic of HEV-3b. The zinc-binding motif and replication catalytic triad of **RdRp** were fully conserved. By contrast, substitutions were found to affect motifs I (0.2%), II (0.8%), III (13.3%), IV (0.9%), V (7.9%), VII (2.8%) and VIII (0.9%). Of the seven previously described ribavirin resistance mutations, the most common were V1479I (67.2%) and G1634RK (10.3%).

**Table 2:** Analysis of previously reported motifs and mutations of ORF1 of HEV-3 genome (references of motifs and mutation are mentioned in the Introduction and listed in full in Supplementary material).

	Functional motif /mutation (Position according to ORF) (Associated with)	Residues (Position beginning each protein)	Substitutions sequences (*n*; %)	Clade/subtype polymorphism
Mtase	Motif I, II, IV (H^65^, DXXXR^113–117^, Y^208^)		0	I^161^ (3efg) S^172^ (3f) S^141^ (3k) S^154^ (3k) S/P^154^ (3c)
	C residues (C^146^-C^153^)		0	
	A27V; N29D (Increased viral load and severity)		0	
	R105H (Decreased viremia)		0	
	F179S (Fulminant hepatitis in HEV-1)	124	A179F (1; 0.2%)	
Y	Cysteine dyad (C^336^-C^337^) (Palmitoylation site)		0	–
	α-helix (LYSWLFE^410–416^) (Inhibit infectivity)		0	
PCP	CRC^457–459^ CTC^481–483^ (Zn^2+^ binding. Loss of activity)		0	A^454^ S^461^C^495^ G^559^ (3efg); D^539^ (3f-A1) R^475^ (3abjk) C^495^ (3m); A^546^ (3b) S^575^ I^576^ T^577^ (3ef)
	C^471^, C^472^ (C471A, C472A suppressed replication)		0	
	H^497^, H^581^ (H497L, H581L suppressed replication)	H^64^, H^148^	YQR^581^ (10; 1.9%)	
	Catalytic dyad C^434^-H^443^ (PCP function)	C^1^-H^10^	S^434^, Y^443^ (2; 0.4%)	
	W module (W^437^-W^476^) (Structural integrity)		0	
X domain	NxxNxxHxxGGG^815–826^ (Appr-1″-pase active site that formed the secondary structure)	19–30	D^826^ (1; 0.2%)	Y^835^ (3chil) A^838^ (3abjk) D^843^ S^960^ (3efg) V^845^ (3f); L^888^ (3ef) D^848^ D^926^ (3chilm)
	(I^860^-I^861^/L^895^-L^896^) (ORF3 interaction, life-cycle)		0	
	N^815^, A^816^ (Metal binding sites)		0	
	GGGLCHAF^824–831^ (Metal binding sites)	28–35	D^826^, I^827^, LR^829^ (5; 0.9%)	
	P^927^ (Metal binding sites)	131	L^927^ (75; 14.1%) (3c)	
Helicase	Motif I (Walker A) (GVPGSGKS^985–992^) (ATPase activity, K^991^ mutation abolish it)	16–23	D^988^, Y^989^, E^990^, THE^991^, T^992^ (3; 0.6%)	I^1032^ (3k) L^1032^ (3h) T^1032^ (3m) A^1032^ (3i, 3ef) G^1032^ (3g)
	Motif Ia (DVVVVPTREL^1001–1010^) (ATPase activity)	32–41	H^1010^ (1; 0.2%)	
	Motif II &Walker B: GRRVVI^1033–1038^/DEAP^1039–1042^ (D residue interacts with Mg^2+^, NTPase activity)	64–73	C^1034^, I^1037^ (3; 0.6%)	
	Motif III (HLLGDPNQ^1060–1067^) (ATPase activity)	91–98	L^1060^, H^1067^ (2; 0.4%)	
	Motif IV (THRCPA^1095–1100^)		0	
	Motif V (TVHEAQGATFTETTI^1149–1163^)	180–194	T^1153^,Y^1158^, DKV^1160^,V^1163^ (34;6.4%)	
	Motif VI (VALTRHTEK^1182–1190^) (binding, hydrolysis)	213–221	G^1189^, R^1190^ (17; 3.2%)	
	L1110F (Fulminant hepatitis in HEV-1)	151	MQRSV^1110^ (53; 9.9%) (3e, 3f-A2)	
	V1120I (Fulminant hepatitis in HEV-1)	161	I^1120^ (277; 51.9%) TS^1120^ (41; 7.7%)	
	V1208A (Increased virulence in HEV-3)	239	A^1208^ (13; 2.4%) (3b) IT^1208^ (386; 71.1%)	
RdRp	C^1247^ (Zn^2+^ binding motif)		0	G^1342^ S^1455^ I^1458^ A^1608^ l^1702^ (3efg) F^1449^ (motif III) (3e) I^1688^ (3abjk) D^1451^ (motif III) (3m) M^1702^ (3m)
	Motif I (KDCNKFT^1393–1399^)	177–183	N^1393^ (1; 0.2%)	
	Motif II (SAWSKTFCALFGPWFR^1414–1429^)	198–213	R^1416^, L^1420^ (4; 0.8%)	
	Motif III (FYGDAYEESVF^1444–1454^)	228–238	L^1444^, S^1448^, F^1449^, DGK^1450^, DG^1451^, T^1452^, M^1453^, L^1454^ (71; 13.3%)	
	Motif IV (NDFSEFDSTQNN^1469–1480^)	253–264	S^1479^, D^1480^ (5; 0.9%)	
	Motif V (KHSGEPGTLLWNTVW^1525–1539^)	309–323	S^1531^, I^1538^ (42; 7.9%)	
	GDD^1560–1562^ (Replication catalytic triad)		0	
	Motif VII (PIGLYAG^1593–1599^)	377–383	S^1593^, MV^1594^, FH^1597^ (15; 2.8%)	
	Motif VIII (LPDVVRFAG^1609–1617^)	393–401	D^1611^, I^1612^ (5 0.9%)	
	Y1320H, K1383N, D1384G, K1398R, V1479I, Y1587F, G1634R/K (Ribavirin treatment failure)	114, 177, 178, 192, 273, 381, 428	FHN^1320^ (3; 0.6%); N^1383^ (1; 0.2%); I^1479^ (358; 67.2%); FH^1587^ (2; 0.4%); RK^1634^ (55; 10.3%)	

Table 3 and Figure 2 summarize the ORF2 and ORF3 analysis of the HEV-3 genome. The **ORF2** second start codon had 0.2% of substitutions, while the first codon was fully conserved. Glycosylation sites S137 and T562 were mutated in 0.6 and 2.1%, respectively. Residues whose changes were related to reduced replication and infectivity were mutated in 0.2% (F51L), 0.4% (T59A) and 0.2% (S390L) of analyzed sequences. Of the previously described HEV-3 neutralization epitopes, the P491 epitope did not appear to be mutated in any of the sequences. The remaining epitopes detailed in Table 3 were mutated (0.2%–1.7%). In the case of the epitopes described in HEV-1 and HEV-4, whose mutation frequencies are detailed in Table 3, the V606A substitution was present in 96.2% and L613T was represented in 99.1% of HEV-3 sequences. The first and second start codons of **ORF3** were both fully conserved, and a mutation in the third codon (M10A, 0.2%) was also noted. Position S80, which has been associated with the ORF2–ORF3 interaction, had S80LPF in 3.6% of its sequences. Finally, PSAP motifs involved in replication had a high substitution rate (mainly the first one motif) ranging from 0.2% to 25.7% in the residues involved. One sequence harbored mutations in both PSAP motifs.

**Table 3:** Analysis of previously reported motifs, mutations and neutralization epitopes (NE) of ORF2 and ORF3 of HEV-3 genome (references of motifs and mutation are mentioned in the Introduction and listed in Supplementary material).

	Functional motif/mutation (Associated to)		Substitutions sequences (*N*; %)	Clade/subtype polymorphism
ORF2	First AUG (M^1^) Capsid-associated ORF2		0	A^86^, T^264^, T^426^, T^593^, I^595^ (3abjk) T^86^ (3h, 3l) A/V^80^ (3f)
	Second AUG (M^16^) ORF secreted form		I^16^ (1; 0.2%)	
	Glycosylation sites: NLS^137^ NLT^310^ NTT^562^ (N562QDPY affects ORF2 dimerization and HEV infectivity)		DFK^137^ (3; 0.6%)	
			D^562^ (11; 2.1%)	
	F51L (Decrease replication and infectivity)		L^51^ (1; 0.2%)	
	T59A (Decrease replication and infectivity)		A^59^ (2; 0.4%) N^59^ (1; 0.2%)	
	S390L (Decrease replication and infectivity)		L^390^ (1; 0.2%)	
	D430, L433, Y485, and R512	NE in HEV-1	0	
	E479		GK^479^ (2; 0.4%)	
	D496		NY^496^ (4; 0.8%)	
	I529		VT^529^ (10; 1.9%)	
	K534		R^534^ (1; 0.2%)	
	H577		R^577^ (1; 0.2%)	
	R578		P^578^ (1; 0.2%)	
	V606A		A^606^ (513; 96.2%) GT^606^ (2; 0.4%)	
	S487	NE in HEV-3	PT^487^ (2; 0.4%)	
	S488		P^488^ (2; 0.4%)	
	T489		A^489^ (1; 0.2%)	
	P491		0	
	D496		NY^496^ (4; 0.8%)	
	Y561		F^561^ (1; 0.2%)	
	T564		AINS^564^ (4; 0.8%)	
	T585		N^585^ (1; 0.2%)	
	T586		HP^586^ (2; 0.4%)	
	P592		LI^592^ (7; 1.3%)	
	L613T	NE in HEV-4	T^613^ (537; 99.1%) AI^613^ (3; 0.6%)	
	L477T (L476T)		0	
	E549	NE in HEV-1 and 4	0	
	K554		R^554^ (12; 2.3%)	
	T497	NE in HEV-1 and 3	A^497^ (2; 0.4%)	
	G591	NE in HEV-1, 3 and 4	AES^591^ (9; 1.7%)	
ORF3	First AUG (M^1^)		0	–
	Second AUG (M^3^)		0	
	Third AUG (M^10^) (True initial ORF3 protein)		A^10^ (1; 0.2%)	
	S80A (S79A) (V66G-ORF2) (Assembly and ORF2/3 interaction)		LFP^80^ (19; 3.6%)	
	PSAP motif (first) PSAP^95–98^ (Mutations in both motifs decrease replication)		HLR^95^ (8; 1.5%)	
			LP^96^ (137; 25.7%)	
			VSG^97^ (73; 13.7%)	
			QHLR^98^ (21; 3.9%)	
	PSAP motif (second) PSAP^104–107^ (Mutations in both motifs decrease replication)		NRC^105^ (7; 1.3%)	
			V^106^ (1; 0.2%)	

### Clade/subtype-associated polymorphisms

Some amino acid polymorphisms were characteristic of different clades/subtypes, by which we mean that up to 90% of sequences of the clade/subtype harbor a particular polymorphism (Supplementary Table 1; Tables 2, 3). In **MTase**, position 161I was characteristic of HEV-3efg, while 172S was specific to subtype HEV-3f, 141S of HEV-3k and 154S of HEV-3k and HEV-3c. In **PCP** 454A, 461S, 495C, 559G, 575S, 576I, and 577T are characteristic of HEV-3efg/ef, and 475R characterizes HEV-3abjk. The 495C, 546A, and 539D residues were characteristic of HEV-3m, HEV-3b and HEV-3f-A1, respectively. No clade or subtype polymorphisms were described in the **Y domain**. In the **X domain**, 838A was associated with HEV-3abjk; 843D, 888l, and 960S were associated with HEV-3efg/ef; and 835Y, 848D and 926D were linked to HEV-3chilm/chil. 845V is characteristic of HEV-3f subtype. In **Hel**, position 1,032 featured a different amino acid in each of the HEV-3 subtypes (Supplementary Table 1). In the case of **RdRp**, 1342G, 1455S, 1458I, 1608A, and 1702l are characteristic of the HEV-3efg clade, as 1688I is of HEV-3abjk. Furthermore, 1449F (motif III) was found in HEV-3e and 1451D (motif III), and 1702M was present in HEV-3m. HEV-3abjk presented several characteristic mutations in **ORF2**: 86A, 264T, 426T, 593T, and 595I. 86T was found in HEV-3hl subtypes and 80AV was present in the HEV-3f subtype. **ORF3** contained no clade or subtype polymorphisms.

## Discussion

HEV is considered an emerging infection in Europe, and is known to be of great clinical relevance in immunocompromised patients. Disease severity depends on host immune status (von Felden et al., 2019) and it has been suggested that this might be associated with specific nucleotide substitutions (Inoue et al., 2009). In fact, HEV is composed of a wide range of virus genotypes and subtypes whose relationships between genetic characteristics and clinical manifestations are yet to be fully explored. The HEV genome is not completely understood, and the PCP, Y domain and HVR still await a clear functional assignment. In this context, we analyzed the HEV genome in depth.

First, we analyzed the variability of the HEV genome and, for the first time, examined the entropy and identified significant differences among genotypes; in short, we found the entropy of zoonotic genotypes to be higher than that of non-zoonotic genotypes in the X domain, RdRp, ORF2 and ORF3. Entropy reflects the diversity of circulating viruses and is somehow related to epidemiological behavior, including the emergence of new variants, as suggested by studies of SARS CoV-2 epidemic in India (Santoni et al., 2022) and mumps outbreaks in Spain (Gavilan et al., 2022). The greater diversity of circulating zoonotic viruses might be related to the more frequent introduction of variants due to the broader diversity of hosts or the wider geographical distribution of HEV-3 and HEV-4 genotypes. It is striking that no significant differences were found among the HEV-3 clades, suggesting that different HEV-3 clades might have similar epidemiological behavior. When analyzing entropy differences among the HEV genome proteins, excluding HVR, we found the Y domain to be extremely highly conserved, suggesting that this protein has a crucial role in the viral cycle.

Second, we analyzed the previously described motifs and mutations in HEV-3 ORF1, of which the completely conserved Mtase and Y domain motifs (Rozanov et al., 1992; Cao et al., 2018) were of particular note. By contrast, the PCP essential H581 residue and catalytic dyad C434-H443 (Parvez, 2013) exhibited amino acid changes in 1.8% and 0.4% of sequences, respectively. This finding is in line with the still poorly defined functions of the PCP region. The X domain is present in the *Hepeviridae*, *Togaviridae* and *Coronaviridae* families, where it is considered a putative IFN antagonist that modulates the host immune response as well as being associated with viral pathogenesis and replication (Li et al., 2016). In addition, both HEV X domain and polyproline region heterogeneity were proposed as being associated with viral persistence (Lhomme et al., 2014). Interestingly, our results show that the HEV-3 X domain has several polymorphic amino acids that are associated with clades, an observation that might be consistent with the recently reported clade differences in clinical behavior (Schemmerer et al., 2022). Additionally, it has recently been suggested that the conserved macrodomain is a potential therapeutic target for coronavirus and alphavirus (Leung et al., 2022). The entropy of the X domain is among the highest in our study, so the treatment target of this genome region might not be an option for HEV-3. We found sequences with mutations in different residues of all Hel motifs except motif IV. Motifs I, II and VI mediate binding and hydrolysis of NTP, while III and VI are involved in coupling ATPase activity to helicase function (Hall and Matson, 1999). Also in the Hel, point mutations have been described in positions 1,110, 1,120, and 1,208 that are related to increased virulence and/or fulminant hepatitis (Takahashi et al., 2009; Devhare et al., 2014). These point mutations are frequently observed in the studied sequences (especially I^1120^, which occurs in 52.1% of sequences). Interestingly, the 1,110 residue constitutes a polymorphism typical of HEV-3e and the recently described HEV-3f-A2 (Munoz-Chimeno et al., 2022), while the 1,208 residue is typical of HEV-3b. This finding suggests a possible association of the subtype with severity of the disease. Although information about the clinical course of cases is not available, most of the complete genomes available in the GenBank database presumably correspond to clinical cases. It would be interesting to carry out prospective studies with clinical information to assess whether the presence of these mutations is linked to the clinical course. The study of these mutations might be useful for patient management and prediction outcome. RdRp motifs III, V and VII also had high mutation rates. Motif III includes residues that are polymorphisms of HEV-3e (F1449) and HEV-3m (D1451). Ribavirin is currently used as first-line antiviral therapy to treat severe or chronic HEV infection (Raji et al., 2022). *In vitro* studies have identified associations between some RdRp point mutations and treatment failure (Debing et al., 2014, 2016; Borkakoti et al., 2016, 2017; Todt et al., 2016). These mutations are very frequent in the analyzed sequences, especially 1479I and 1634R/K, which are present in 67.4% and 10.0% of sequences, respectively. 1479I is present in 100% of HEV-3efg, HEV-3l, HEV-3m, and HEV-3k, while 1634R occurs in almost 100% of HEV-3e. This means that, in the case of ribavirin treatment, it might be necessary to have subtype information in order to predict antiviral response, especially when a long-term treatment is needed.

Third, two initial codons have been described in HEV ORF2 that are of uncertain significance in the viral cycle (Yin et al., 2018). The second initial codon could give rise to a strategy of immune toleration similar to that of hepatitis B virus (HBV). HBV generates “e” protein using an alternative to the core protein initial codon, the action being related to a milder immune response and less cellular damage. Mutation of this initial codon or the generation of a stop codon causes a severe and rapidly evolving HBV infection (Malik et al., 2018). One of the studied sequences has a mutation in this alternative codon. It would be very interesting to examine the relationship between the presence of these mutations and the clinical course of the infection. Neutralization epitopes have been described for HEV-1, HEV-3, and HEV-4 (Gu et al., 2015; Ikram et al., 2018) in 27 residues of ORF2, of which 430, 433, 476, 477, 485, 491, 512, and 549 had no mutations in the HEV-3 sequences studied. The remaining 19 sequences had mutation rates between 0.2% and 99.1%. These results suggest that immune epitopes are highly variable and highlight the importance of developing a pan-genotypic vaccine (Nan et al., 2018).

Finally, no mutation was found in the first and second codons of HEV ORF3, although one was found in one sequence in the third codon. Mutation in assembly interaction serine ORF2-ORF3 (S80) was detected in 3.5% of sequences to L, F and P. Although amino acid change described as affecting assembly was S80A due to the loss of phosphorylated S (Nagashima et al., 2011), none of the amino acids found are known to be phosphorylated, so assembly could also be impaired. Amino acids in the PSAP motif had a high substitution rate, being more frequent in the first PSAP motif (44.8% sequences with substitutions) than in the second (1.5%). One sample featured a mutation in both PSAP motifs. PSAP motifs are required for transport machinery and promote HEV envelopment and exit *via* the exome pathway. At least one of the two PSAP motifs is needed to form the membrane-associated HEV particles (Nagashima et al., 2011). According to our results, the second PSAP motif appears to be more important in this respect than the first.

This study, together with the HVR analysis previously reported (Munoz-Chimeno et al., 2020), constitute the first comprehensive genome analysis of HEV-3. The aim was to provide not only to improve our knowledge of the genome, but also to establish the basis for future genotype-to-phenotype analyses. HEV-3 is a virus with low pathogenicity in immunocompetent patients, but the viral features associated with its severity have not been explored in depth. Our results reveal important genetic differences in the studied genomes that sometimes affect significant viral structures, and that constitute clade or subtype polymorphisms that may affect the clinical course or treatment efficacy.

## Data availability statement

The original contributions presented in the study are included in the article/Supplementary material, further inquiries can be directed to the corresponding author.

## Ethics statement

The sequences obtained in this study were based on routine HEV testing, did not involve any additional sampling or tests, and irreversibly anonymized RNA extracts were used, so specific ethical approval was not required.

## Author contributions

MM-C performed the laboratory assays and sequence analysis and wrote the article. VR-P and MG-L helped carry out the laboratory assays. AA directed and coordinated the study and the production of the manuscript. All authors contributed to the article and approved the submitted version.

## Funding

MM-C was funded by CIBERESP.

## Conflict of interest

The authors declare that the research was conducted in the absence of any commercial or financial relationships that could be construed as a potential conflict of interest.

## Publisher’s note

All claims expressed in this article are solely those of the authors and do not necessarily represent those of their affiliated organizations, or those of the publisher, the editors and the reviewers. Any product that may be evaluated in this article, or claim that may be made by its manufacturer, is not guaranteed or endorsed by the publisher.

## Footnotes

^1^ https://www.megasoftware.net/
