# Supplementary material for: Hepatitis E genotype 3 genome: A comprehensive analysis of entropy, motif conservation, relevant mutations, and clade-associated polymorphisms
Source: Front Microbiol. 2022 Oct 6;13:1011662. doi: 10.3389/fmicb.2022.1011662 (PMC9582770; doi:10.3389/fmicb.2022.1011662)
Supplement: Supplementary file 1 [file Data_Sheet_1.docx]

Data Sheet 1. GenBank accession numbers of sequences used in the study. References sequences include the subtype.

GENOTYPE 1:

DQ459342, MH918640, AF051830, AF076239, AF459438, DQ459342, JF443718, JF443719, JF443720, LC061267, M73218_1a, MH504157, AF444002, AF444003, D11092, D11093, JQ655734, L08816_1b, M94177, NC_001434, JF443717, X98292_1c, AY230202_1d, AY204877_1e, AB720034, AB720035, JF443721_1f, JF443722, JF443723, JF443724, JF443725, JF443726, MH074880, MH504154, MH504162, MH991993, MH991994, MH991995, MH991996, MH991997, MH991998, MH991999, MH992000, MH992001, MH992002, MH992003, MH992004, MH992005, MH992006, MH992007, MH992008, MH992009, MH992010, MH992011, MH992012, MH992013, LC225387_1g, LC314155, LC314156, LC314157, LC314158, MH504155, MH504156, MH504158, MH504159, MH504160, MH50416, MH504163 and MN401238.

GENOTYPE 3:

KF303502, AB074918, AB074920, AB089824, AB481228, AB591734, AB630970, AF060668, AF060669, AX181807, AX181808, AX181882, AX181883, AY575857, AY575858, AY575859, BD378054, BD378055, BD378126, BD378127, FJ426403, FJ426404, AF082843_3a, HQ389543, HQ389544, HQ709170, HW532736, HW532737, JC087002, JC087006, JN564006, JN837481, JQ679013, JQ679014, KJ507955, KT447526, KT447528, KT727028, MG833836, AB073912, AB222184, KT633715_3CHINA, FJ527832, KJ507956, AB091394, AB189070, AB189071, AB189072, AB189073, AB189074, AB189075, AB222182, AB222183, AB236320, AB246676, AB291951, AB291952, AB291953, AB291954, AB291955, AB291956, AB291957, AB291960, AB291962, AB291963, AB301710, AB362839, AB362840, AB362841, AB362842, AB362843, AB369691, AB425830, AB425831, AB437316, AB437317, AB437318, AB443623, AB443624, AB443625, AB443626, AB443627, AB481229, AB591733, AB593690, AB630971, AB698071, AP003430_3b, LC126331, LC126332, LC386855, LC439299, AY115488_3j, AB740232, AB369689_3k, LC131066, LC176492, LC176493, KP294371_3GERMANY, MF959764_3ITALY, MW355317, MW355383, MF959765_3ITALY, MK390971_3ITALY, LC260517_3JAPAN, FJ705359_3c, KC618402, KC618403, KT159771, KU176129, KX172133, MF444042, MF444049, MF444065, MF444072, MF444085, MF444106, MF444111, MF444115, MF444122, MF444128, MF444143, MG783569, MG783570, MW355217, MW355218, MW355220, MW355221, MW355224, MW355226, MW355229, MW355231, MW355232, MW355233, MW355234, MW355235, MW355245, MW355256, MW355262, MW355263, MW355267, MW355268, MW355269, MW355270, MW355271, MW355273, MW355277, MW355278, MW355279, MW355280, MW355282, MW355283, MW355284, MW355286, MW355287, MW355293, MW355294, MW355295, MW355297, MW355298, MW355300, MW355303, MW355304, MW355309, MW355312, MW355313, MW355314, MW355316, MW355320, MW355321, MW355323, MW355327, MW355328, MW355332, MW355335, MW355338, MW355345, MW355347, MW355357, MW355360, MW355362, MW355364, MW355365, MW355367, MW355370, MW355372, MW355374, MW355378, MW355379, MW355385, MW355386, MZ289076, MZ289133, MF444043, MF444114, MW355264, MW355334, MW355341, JQ013794_3h, KU176131, KU176132, MF444037, MF444056, MF444077, MF444110, MF444120, MF444136, MW355246, MW355302, MW355307, AB290312, KY780957, MF346772, MF346773, MG573193, FJ998008_3i, JQ953664_3l, KY766999, MF444131, MG674164, MW355275, KU513561_3m, KU176130, MF444089, MZ289090, MZ289096, MZ289119, MF444030, MW355236, MZ289082, MZ289100, MZ289103, MZ289113, AB290313_3MONGOLIA, AB248520, AB248522, AB291958, AB481226, AB780450, AB780451, AB780452, AB780453, FJ998015, AB248521_3e, HM055578, JQ013795, JQ026407, JQ953665, KF922359, KP698919, MF444109, MF444141, MH184579, MH184580, MH184581, MH184582, MH184583, MH184584, AB291961, AB850879, EU375463, EU495148, EU723512, EU723513, EU723514, EU723515, EU723516, FJ653660, FJ956757, AB369687_3f-A1, JN906974, JN906975, JN906976, JQ953666, KC166967, KC166968, KC166969, KC166970, KC166971, KT581448, KT591532, KT591533, KY232312, KY232313, LC055972, LC055973, LC164712, MF444027, MF444028, MF444029, MF444032, MF444034, MF444035, MF444036, MF444038, MF444039, MF444040, MF444041, MF444045, MF444046, MF444047, MF444048, MF444050, MF444051, MF444052, MF444054, MF444055, MF444057, MF444058, MF444059, MF444061, MF444066, MF444067, MF444068, MF444069, MF444070, MF444073, MF444076, MF444078, MF444079, MF444081, MF444082, MF444084, MF444087, MF444088, MF444090, MF444092, MF444093, MF444094, MF444095, MF444096, MF444097, MF444098, MF444100, MF444101, MF444102, MF444103, MF444104, MF444105, MF444107, MF444108, MF444112, MF444113, MF444116, MF444117, MF444123, MF444124, MF444125, MF444127, MF444129, MF444130, MF444132, MF444133, MF444134, MF444135, MF444137, MF444138, MF444139, MF444140, MF444142, MW355219, MW355223, MW355227, MW355228, MW355230, MW355237, MW355238, MW355239, MW355240, MW355241, MW355242, MW355244, MW355247, MW355248, MW355249, MW355250, MW355251, MW355252, MW355253, MW355254, MW355255, MW355257, MW355258, MW355259, MW355260, MW355261, MW355265, MW355266, MW355272, MW355274, MW355276, MW355285, MW355289, MW355291, MW355292, MW355296, MW355299, MW355301, MW355305, MW355306, MW355308, MW355310, MW355311, MW355315, MW355318, MW355322, MW355324, MW355325, MW355326, MW355329, MW355330, MW355331, MW355333, MW355336, MW355337, MW355339, MW355340, MW355342, MW355343, MW355346, MW355348, MW355349, MW355350, MW355351, MW355352, MW355353, MW355354, MW355355, MW355356, MW355358, MW355361, MW355363, MW355371, MW355373, MW355375, MW355376, MW355380, MW355381, MW355384, MW355387, MW355388, MW355389, MW355390, MZ289077, MZ289078, MZ289080, MZ289081, MZ289085, MZ289086, MZ289087, MZ289088, MZ289089, MZ289091, MZ289092, MZ289097, MZ289098, MZ289099, MZ289102, MZ289105, MZ289106, MZ289107, MZ289108, MZ289109, MZ289111, MZ289114, MZ289115, MZ289116, MZ289118, MZ289120, MZ289123, MZ289124, MZ289125, MZ289126, MZ289127, MZ289128, MZ289129, MZ289130, MZ289131, MZ289132, MZ289134, MZ289136, MZ289138, MZ289139, MZ289140, MZ289141, MZ289142, MZ289143, MZ289144, MZ289145, MZ289146, MZ289147, MZ289148, MF444091_3f-A2, MF444053, MF444075, MW355243, MW355281, MW355290, MW355344, MW355359, MW355366, MW355368, MW355377, MZ289079, MZ289083, MZ289084, MZ289093, MZ289094, MZ289095, MZ289101, MZ289104, MZ289110, MZ289112, MZ289117, MZ289121, MZ289135, MZ289137, MZ289149, MW355382, EU360977_3f-B, KT447527, KT581443, KT581444, KT581445, KT581446, KT581447, KU747141, KU747142, KU980235, MW355319, MW355369, MZ289122 and AF455784_3g.

GENOTYPE 4:

AB369688_4JAPAN, MK410048_4CHINA, AB197673_4a, AB197674, EF077630, EU366959, FJ763142, GU119960, HQ634346, JQ655733, KC492825, KC692453, LC037955, MK410045, MK410046, MK410047, MK410049, MK410050, MK410051, MK410052, MK410053, AB253420, AB291964, DQ279091_4b, EU676172, JX855794, LC0422320, LC428039, LC436449, LC436450, AB074915_4c, AB074917, AB080575, AB091395, AB097811, AB097812, AB099347, AB193176, AB193177, AB193178, AB200239, AB220971, AB220972, AB220973, AB220975, AB220976, AB220977, AB220978, AB220979, AB291959, AB291965, AB291966, AB291967, AB291968, AB481227, LC022745, LC387631, AJ272108_4d, AY594199, FJ610232, GU206559, GU361892, JQ655736, KC163335, KF176351, KX531115, KX827238, MK410044, AY723745_4e, AB220974_4f, AB108537_4g, AB698654, LC387632, GU119961_4h, JQ655735, JQ740781, KF736234, KJ155502, KM253769, KR872414, KR872415, KR872416, KR872417, KU356182, KU356183, KU356184, KU356185, KU356186, KU356187, KU356188, KU356189, AB369690_4i, AB521805, AB521806, AB602439, AB602440, AB909124, AB909125, DQ450072, EF570133, HM439284, JF915746 and JQ993308.
