# Supplementary material for: Hepatitis E genotype 3 genome: A comprehensive analysis of entropy, motif conservation, relevant mutations, and clade-associated polymorphisms
Source: Front Microbiol. 2022 Oct 6;13:1011662. doi: 10.3389/fmicb.2022.1011662 (PMC9582770; doi:10.3389/fmicb.2022.1011662)
Supplement: Supplementary file 2 [file Table_1.DOCX]

**Supplementary Table 1**. Detailed clade/subtype polymorphisms in HEV-3 proteins.

|  | **Protein / Domain** | | **Protein position** | **3abjk (n=99)** | | | **3chilm (n=135)** | | | **3efg (n=300)** | | | |  |
| --- | --- | --- | --- | --- | --- | --- | --- | --- | --- | --- | --- | --- | --- | --- |
|  |  |  |  | **Subtype Clade** | **AA** | **Frequency (%)** | **Subtype Clade** | **AA** | **Frequency (%)** | | **Subtype Clade** | **AA** | **Frequency (%)** | |
| **ORF1** | **Mtase** | | 81 | 3k | S | 100 (5/5) | 3chilm | A | 100 (135/135) | | 3efg | A | 100 (300/300) | |
|  |  |  |  | 3abj | A | 96.81 (91/94) |  |  |  |  |  |  |  |  |
|  |  |  | 154 | 3k | S | 100 (5/5) | 3c | S/P | 98.92 (92/93) | | 3efg | A | 96.98 (290/299) | |
|  |  |  |  | 3abj | A | 92.47 (86/93) | 3hilm | A | 97.14 (34/35) | |  |  |  |  |
|  |  |  | 161 | 3abjk | V | 100 (98/99) | 3chilm | V | 95.56 (129/135) | | 3efg | I | 96.31 (296/298) | |
|  |  |  | 172 | 3abjk | A | 100 (93/93) | 3chilm | A | 100 (135/135) | | 3f | S | 99.63 (272/273) | |
|  |  |  |  |  |  |  |  |  |  |  | 3e. 3g | A | 100 (25/25) | |
|  | **PCP** | | 454 | 3abjk | S | 97.98 (97/99) | 3chilm | S | 100 (135/135) | | 3efg | A | 96 (288/300) | |
|  |  |  | 461 | 3abjk | T | 98.99 (98/99) | 3chilm | T | 100 (135/135) | | 3efg | S | 98.67 (296/300) | |
|  |  |  | 475 | 3abjk | R | 97.98 (97/99) | 3chilm | K | 97.78 (132/135) | | 3efg | K | 100 (299/299) | |
|  |  |  | 495 | 3abjk | H/Q | 95.96 (95/99) | 3m | C | 100 (12/12) | | 3efg | C | 98.33 (295/300) | |
|  |  |  |  |  |  |  | 3chil | H/Q | 96.75 (119/123) | |  |  |  |  |
|  |  |  | 509 | 3k | P | 100 (5/5) | 3chilm | Q | 95.56 (129/135) | | 3efg | P | 97 (291/300) | |
|  |  |  |  | 3j | R | 100 (1/1) |  |  |  |  |  |  |  |  |
|  |  |  |  | 3b | Q | 90.74 (49/54) |  |  |  |  |  |  |  |  |
|  |  |  | 539 | 3abjk | H/Q | 98.99 (98/99) | 3chilm | H | 94.07 (127/135) | | 3f-A2, 3f-B | H | 97.50 (39/40) | |
|  |  |  |  |  |  |  |  |  |  |  | 3f-A1 | D | 97.44 (228/234) | |
|  |  |  |  |  |  |  |  |  |  |  | 3e | H | 95.83 (23/24) | |
|  |  |  | 546 | 3b | A | 100 (54/54) | 3chilm | S | 100 (135/135) | | 3efg | S | 94.64 (283/299) | |
|  |  |  |  | 3a, 3j, 3k | S | 100 (45/45) |  |  |  |  |  |  |  |  |
|  |  |  | 559 | 3abjk | D | 100 (99/99) | 3chilm | D | 97.78 (132/135) | | 3efg | G | 100 (300/300) | |
|  |  |  | 575 | 3abjk | T | 100 (99/99) | 3chilm | T | 100 (135/135) | | 3e, 3f | S | 99.67 (299/300) | |
|  |  |  | 576 | 3abjk | V | 96.97 (96/99) | 3chilm | V | 100 (135/135) | | 3e, 3f | I | 99,67 (299/300) | |
|  |  |  | 577 | 3abjk | V | 88.89 (88/99) | 3chilm | V | 100 (135/135) | | 3e, 3f | T | 92.64 (277/299) | |
|  | **X domain** | | 835 | 3abjk | F | 98.99 (98/99) | 3c, 3h, 3i, 3l | Y | 98.37 (121/123) | | 3efg | F | 98.33 (295/300) | |
|  |  |  |  |  |  |  | 3m | F | 100 (12/12) | |  |  |  |  |
|  |  |  | 838 | 3abjk | A | 100 (99/99) | 3chilm | S | 99.26 (134/135) | | 3efg | S | 99.66 (297/298) | |
|  |  |  | 843 | 3abjk | E | 94.95 (94/99) | 3chilm | E | 98.52 (133/135) | | 3efg | D | 99.33 (297/299) | |
|  |  |  | 845 | 3abjk | I | 97.98 (97/99) | 3chilm | I | 91.11 (123/135) | | 3e, 3g | I | 87.50 (21/24) | |
|  |  |  |  |  |  |  |  |  |  |  | 3f | V | 95.60 (261/273) | |
|  |  |  | 848 | 3abjk | E | 100 (99/99) | 3chilm | D | 99.26 (134/135) | | 3e, 3f | E | 96.30 (286/297) | |
|  |  |  |  |  |  |  |  |  |  |  | 3g | D | 100 (1/1) | |
|  |  |  | 888 | 3abjk | R | 100 (99/99) | 3chilm | R | 99.26 (134/135) | | 3e, 3f | L | 99.66 (297/298) | |
|  |  |  |  |  |  |  |  |  |  |  | 3g | R | 100 (1/1) | |
|  |  |  | 926 | 3abjk | E | 100 (99/99) | 3chilm | D | 98.52 (133/135) | | 3efg | E | 100 (300/300) | |
|  |  |  | 960 | 3abjk | A | 100 (99/99) | 3chilm | A | 99.26 (134/135) | | 3efg | S | 99.67 (299/300) | |
|  | **Helicase** | | 1032 | 3k | I | 100 (5/5) | 3c | T | 88.17 (82/93) | | 3ef | A | 96.30 (286/297) | |
|  |  |  |  |  |  |  | 3h | L | 100 (17/17) | |  |  |  |  |
|  |  |  |  |  |  |  | 3i | A | 100 (1/1) | | 3g | G | 100 (1/1) | |
|  |  |  |  |  |  |  | 3m | T | 100 (12/12) | |  |  |  |  |
|  | **RdRp** | | 1227 | 3abjk | V | 100 (99/99) | 3chilm | V | 100 (135/135) | | 3efg | T/I | 95.99 (287/299) | |
|  |  |  | 1238 | 3a, 3b | G/E | 93.61 (88/94) | 3c | A | 98.92 (92/93) | | 3efg | D | 99.00 (296/299) | |
|  |  |  |  |  |  |  | 3l | A | 100 (5/5) | |  |  |  |  |
|  |  |  |  | 3k | D | 100 (5/5) | 3h, 3i, Untyped | E | 92.00 (23/25) | |  |  |  |  |
|  |  |  |  |  |  |  | 3m | E | 100 (12/12) | |  |  |  |  |
|  |  |  | 1342 | 3abjk | A | 100 (99/99) | 3c | A/T | 100 (93/93) | | 3efg | G | 95.32 (285/299) | |
|  |  |  |  |  |  |  | 3h, 3i, 3l, 3m, Untyped | A | 100 (42/42) | |  |  |  |  |
|  |  |  | 1449 | 3abjk | Y | 100 (99/99) | 3chilm | Y | 99.26 (134/135) | | 3e | F | 100 (24/24) | |
|  |  |  |  |  |  |  |  |  |  |  | 3f, 3g | Y | 99.64 (274/275) | |
|  |  |  | 1455 | 3abjk | A | 98.99 (98/99) | 3chilm | A | 99.26 (134/135) | | 3efg | S | 100 (300/300) | |
|  |  |  | 1458 | 3abjk | V | 100 (99/99) | 3chilm | V | 100 (135/135) | | 3efg | I | 96.99 (290/299) | |
|  |  |  | 1608 | 3abjk | T | 97.98 (97/99) | 3chilm | T | 98.52 (133/135) | | 3efg | A | 99.67 (299/300) | |
|  |  |  | 1644 | 3a, 3b | G | 100 (93/93) | 3chilm | G | 100 (135/135) | | 3e | R | 95.83 (23/24) | |
|  |  |  |  | 3j, 3k | R | 83.33 (5/6) |  |  |  |  | 3f, 3g | G | 90.55 (249/275) | |
|  |  |  | 1688 | 3abjk | I | 90.91 (90/99) | 3chilm | V | 100 (135/135) | | 3efg | V | 87.63 (262/299) | |
|  |  |  | 1702 | 3abjk | V | 94.95 (94/99) | 3c, 3i, 3l, 3m-B, untyped | V | 95.54 (107/112) | | 3efg | L | 94.31 (282/299) | |
|  |  |  |  |  |  |  | 3h | I/E | 88.24 (15/17) | |  |  |  |  |
|  |  |  |  |  |  |  | 3m-A | M | 100 (6/6) | |  |  |  |  |
| **ORF2** | **S domain** | **N-terminal** | 80 | 3abjk | P | 98.99 (98/99) | 3c | P/S | 93.55 (87/93) | | 3e | P | 95.83 (23/24) | |
|  |  |  |  |  |  |  |  |  |  |  | 3f-A1 | A/V | 97.00 (226/233) | |
|  |  |  |  |  |  |  | 3h, 3i, 3l, 3m, Untyped | P | 95.24 (40/42) | | 3f-A2, 3f-B | A | 100 (40/40) | |
|  |  |  |  |  |  |  |  |  |  |  | 3e, 3g | P | 96.00 (24/25) | |
|  |  |  | 86 | 3abjk | A | 98.99 (98/99) | 3c, 3i, 3m, Untyped | S | 98.23 (111/113) | | 3efg | S | 98.67 (296/300) | |
|  |  |  |  |  |  |  | 3h, 3l | T | 100 (22/22) | |  |  |  |  |
|  |  | **C-terminal** | 264 | 3abjk | T | 100 (99/99) | 3chilm | S | 100 (135/135) | | 3efg | S | 100 (300/300) | |
|  |  |  |  |  |  |  |  |  |  |  |  |  |  |  |
|  |  |  |  |  |  |  |  |  |  |  |  |  |  |  |
|  |  |  |  |  |  |  |  |  |  |  |  |  |  |  |
|  |  |  |  |  |  |  |  |  |  |  |  |  |  |  |
|  | **M domain** | | 426 | 3abjk | T | 98.99 (98/99) | 3chilm | A | 98.52 (133/135) | | 3efg | A | 99.00 (297/300) | |
|  | **P domain** | | 593 | 3abjk | T | 98.99 (98/99) | 3chilm | V | 95.52 (128/134) | | 3efg | V | 98.66 (294/298) | |
|  |  |  | 595 | 3abjk | I | 100 (94/94) | 3chilm | V | 90.37 (122/135) | | 3efg | V | 93.94 (279/297) | |
| **ORF3** | **D1** | |  | - | - | - | - | - | - | | - | - | - | |
|  | **D2** | |  | - | - | - | - | - | - | | - | - | - | |
|  | **P1** | |  | - | - | - | - | - | - | | - | - | - | |
|  | **P2** | |  | - | - | - | - | - | - | | - | - | - | |
